# Risk-taking in consumers’ online purchases of health supplements and natural products: a grounded theory approach

**DOI:** 10.1186/s40545-023-00645-x

**Published:** 2023-11-03

**Authors:** Ju-Ying Ang, Guat-See Ooi, Fatimatuzzahra’ Abd.Aziz, Seng-Fah Tong

**Affiliations:** 1 School of Pharmaceutical Sciences, Universiti Sains Malaysia, Gelugor, Pulau Pinang Malaysia; 2 Clinical Research Centre (CRC), Hospital Raja Permaisuri Bainun, Ministry of Health, Ipoh, Perak Malaysia; 3 Department of Family Medicine, Faculty of Medicine, Universiti Kebangsaan Malaysia, Kuala Lumpur, Malaysia

**Keywords:** Risk-taking, Online purchase, Health supplement, Dietary supplement, Natural health product, Traditional and complementary medicine, Theoretical model, Qualitative, Grounded theory, Malaysia

## Abstract

**Background:**

Health supplements and natural products are widely used by the general public to support physical function and prevent disease. Additionally, with the advent of e-commerce, these products have become easily accessible to the general public. Although several theoretical models have been used to explain the use of health supplements and natural products, empirical evidence on how consumers make decisions to purchase online health supplements and natural products remains limited.

**Methods:**

In this study, a grounded theory approach was used to develop a substantive theoretical model with the aim of investigating the decision-making process of consumers when purchasing health supplements and natural products online. Malaysian adult consumers who had purchased these products via the Internet were either purposively or theoretically sampled. A total of 18 virtual in-depth interviews (IDIs) were conducted to elicit participants’ experiences and priorities in relation to this activity. All the IDIs were audio-recorded and transcribed verbatim. The data were analysed using open coding, focus coding and theoretical coding. The analytical interpretations and theoretical concepts were recorded in research memos.

**Results:**

Consumers’ decisions to purchase a health supplement or natural product over the Internet are based on a series of assessments regarding the perceived benefits and risks of this activity, which may be related to the product or the process. In the online marketplace, consumers attempt to choose products, online sellers, sales platforms and/or purchase mechanisms with lower perceived risk, which ultimately enhances their confidence in five elements related to the purchase: (1) product effectiveness, (2) product safety, (3) purchase convenience, (4) fair purchase and (5) online security. Consumers take an acceptable level of risk to purchase these products online, and this acceptable level is unique to each individual and is based on their perception of having control over the potential consequences if the worst-case scenario occurs.

**Conclusions:**

In this study, a substantive theoretical model is developed to demonstrate how consumers decide to purchase online health supplements and natural products by accepting an acceptable level of risk associated with the product or process. The emerging model is potentially transferable to other populations in similar contexts.

## Background

Health supplements and natural products are commonly used by the general public in order to support physical function and to prevent diseases [1], and the prevalence of the use of these products ranges from 22 to 53% across different countries, including the USA, UK, Canada, Korea, Singapore and Malaysia [2, 3]. Specifically, one in four Malaysian adults use health supplements (e.g., vitamins), while one in three of them take some form of natural product (e.g., herbs, herbal remedies) [4]. With the advancement of e-commerce, these products can now be purchased over the Internet, and online purchases are preferred over physical shop purchases due to the convenience, greater product availability and broader brand options [5, 6]. Online purchases of health supplements and natural products and even medication are gaining popularity, particularly due to the recent COVID-19 pandemic [7, 8]. Indeed, the pandemic not only increased the public’s intention to use health supplements in order to prevent COVID-19 [9, 10], but also fuelled unprecedented growth in e-commerce due to national restrictions on public movement [11].

However, the use of health supplements and natural products is not without risk. While consumers typically believe that health supplements and natural products labelled as ‘natural’ are safe and healthy [12], some of these products may be carcinogenic and toxic [13–15]. Additionally, health supplement–drug and herb–drug interactions are two other issues that increase the risk of undesirable clinical outcomes in patients, especially when these products are self-administered by patients without disclosure to their clinicians [16, 17]. In contrast to controlled medicines, which can only be prescribed by a registered doctor or dispensed by a licenced pharmacist, health supplements and natural products are loosely regulated in most jurisdictions [18] and are publicly available over the counter. Before international consensus and a harmonised regulatory framework could be established across countries to regulate the use of health supplements and natural products [19, 20], these products were made available placed on online marketplaces that are easily accessible to consumers worldwide. This availability has magnified the potential risk associated with the use of these products by the general public, as in the online environment, consumers are not guided by healthcare professionals and must make the best decision for themselves. Therefore, it is critical to investigate how consumers navigate the online marketplace to purchase health supplements and natural products.

From the literature, the Health Belief Model (HBM) [21–23] and Protection Motivation Theory (PMT) [24, 25] have been widely used to predict consumers’ use of health supplements, but these models do not provide substantive insight into the online purchasing of this products. Furthermore, consumer behaviour in online purchases has been extensively discussed [26], commonly with the Theory of Reasoned Action (TRA) [27], the Technology Acceptance Model (TAM) [28], the Theory of Planned Behaviour (TPB) [21, 29] and the DeLone and McLean IS success model [30]. These theories outline consumers’ acceptance and experience of new services provided by the internet (such as internet banking, e-commerce, travel online and socialisation) [31], but may not provide a sufficient empirical explanation of the public’s online purchasing behaviour in relation to health-related products specifically. Therefore, we attempt to construct an empirical theoretical framework to explore how consumers decide on online purchases of health supplements and natural products.

## Methods

### Study design

A qualitative study employing the constructivist grounded theory approach was conducted in Malaysia from January 2021 until March 2022. Malaysian consumers who had purchased online health supplements or natural products were recruited for in-depth interviews (IDIs). For the purposes of this study, a health supplement was defined as ‘any product that is used to supplement a diet and to maintain, enhance and improve human body health function’ and that ‘can be formulated in the form of capsule, tablets, powder, or liquid’ [32]. Vitamins, minerals, amino acids, fatty acids, enzymes, probiotics and other natural substances are examples of health supplements, whereas natural products include traditional medicines, herbal products, herbal remedies, and homoeopathic medicine [33]. Only orally administered health supplements and natural products were included in this study.

### Participants and sampling method

The target population of this study was Malaysian consumers aged 18 years or above who had purchased health supplements or natural products from the Internet at least once. Those with intellectual impairment or who were unable to converse in English, Bahasa Malaysia, or Mandarin were excluded from the study. The initial recruitment was conducted through the social contacts of the researchers. In order to capture a high level of variation in online purchase experiences, the participants were purposively sampled from different demographic backgrounds.

Following the 16th IDI, a substantive model was drafted, with ‘taking an acceptable level of risk’ suggested as the core category. According to the model, consumers perceive certain benefits and risks when considering purchasing supplements online. With this draft model in mind, the researchers asked the following questions: ‘How does risk-taking differ across individuals?’ and ‘Do people with critical or chronic illnesses perceive a greater benefit in online supplements, and does this later influence their decisions in online purchases?’ When the researchers returned to the data, they discovered that the majority came from participants who were either healthy or did not have a critical illness. The research team was, thus, unsure about the impact of critical and chronic illness on online supplement purchase decisions. As a result, the research team decided to continue with theoretical sampling by recruiting another two participants with critical or chronic illnesses in order to collect more insightful data that would aid in the final conceptualisation of the theoretical model. Theoretical saturation was achieved after a total of 18 IDIs, as at this point, no new category properties, categories, or new connections between categories were identified [34].

### Data collection

Since face-to-face contact was not encouraged during the study period due to the ongoing COVID-19 pandemic in Malaysia [35], all the IDIs were conducted virtually apart from one, which was conducted over the phone and audio-recorded using a phone recorder. The virtual IDIs were carried out by hosting virtual conversations using a cloud-based video communication application (Zoom) that complies with the Health Insurance Portability and Accountability Act of 1996 (HIPAA) and ensures data confidentiality [36, 37]. The camera functions of both the participant and the interviewer were turned off during the virtual IDI, and the IDI was only audio-recorded without video recording. The audio recording was saved directly to the local device, which was password-protected, rather than being stored in the cloud. Permission for the audio recording of the interviews was obtained from all the participants prior to data collection.

All of the IDIs were conducted by a researcher with a background in clinical research who has received training in qualitative research and is fluent in English, Bahasa Malaysia and Mandarin. The interviewer identified herself to the participants as a postgraduate student conducting a study to fulfil the requirements of the Master of Science degree, and no relationship was established between the interviewer and the participants prior to the IDIs.

During the IDI, participants were allowed to speak in their preferred language, which could be English (8 IDIs), Bahasa Malaysia (3 IDIs), or Mandarin (7 IDIs). In the IDIs, a data collection sheet was used to document the participants’ demographic information, and a semi-structured interview guide was used to elicit information regarding the participants’ online purchase experience, influences from family and friends and perceptions of online purchases of health supplements and natural products. Participants were probed during IDIs regarding specific details and experiences to elicit more accurate and detailed responses. No repeat interviews were conducted, and any data gaps that arose after the initial analysis were explored in the subsequent IDIs. The data collection continued until theoretical saturation.

All the IDIs were transcribed in their original language and cross-checked by another researcher to ensure transcription accuracy [38]. The transcriptions were first managed in Microsoft Word before being imported into the qualitative data analysis software (NVivo 12, QSR International Pty Ltd, 2018) for coding. All the data were anonymised, and each participant was represented by a unique code. To avoid meaning loss due to translation, the non-English (Bahasa Malaysia or Mandarin) transcripts were kept as long as possible in in their source languages [39]. All transcripts were coded in their original languages with only selected verbatims translated into English at the point of analysis [40]. Only one researcher, who is proficient in both the target and source languages, conducted translation in this study to ensure conceptual congruency throughout the translation process [41]. Besides, translations were done with care to preserve the essential meaning of the original verbatims, and the translated verbatims were cross-check by other researchers who are also competent in target and source languages, to ensure translational appropriateness [40]. Any disagreements in translation were discussed and resolved by consensus among the researchers, and the final version of translation was produced.

### Data analysis

Data analysis began after the completion of the first IDI and was carried out concurrently with the data collection. The researchers read the transcripts several times in order to become adequately immersed in the data. One researcher carried out the coding, and another researcher cross-checked the codes. Any coding disagreements were settled through discussion and consensus among the researchers. Initially, open coding was carried out using line-by-line analysis, and the ‘actions’ identified from the data were coded using gerunds. Subsequently, focused coding and theoretical coding were conducted, guided by the analytic direction established in the initial analysis. At this stage, the most significant or frequent initial codes were identified to thoroughly categorise the data.

At each level of analysis, constant comparison (between data, between incidents, and between interviews) was undertaken to identify similarities and differences in the actions and experiences of the participants, and this process led to the emergence of the categories and processes. Ultimately, a core category with the greatest explanatory relevance and highest potential for linking all other categories together through theoretical coding was identified. Moreover, research memos were also written at every stage of the data analysis to record the theoretical concepts, analytical interpretations and frames of relationships and experiences. The researcher's reflexivity was reinforced through debriefing sessions with fellow researchers experienced in the grounded theory research method. In these sessions, the study findings underwent scrutiny, allowing for the identification and rectification of assumptions and subjectivity.

### Ethical considerations

This study received ethics clearance from the Medical Research Ethics Committee, Ministry of Health Malaysia (MREC, Ref. No.: KKM/NIHSEC/P20-1774(4)), and the Human Research Ethics Committee, Universiti Sains Malaysia (HREC, USM/JEPeM/20120641). Prior to recruitment, eligible individuals were informed about the study’s objectives, procedure and the use of audio recording. Participation in the study was completely voluntary, and only those who provided written informed consent were recruited. Throughout the study, data confidentiality was maintained.

Upon the completion of an IDI, each participant was provided with an electronic brochure with information about the safe steps to take when purchasing health products online, which was provided by the Pharmaceutical Services Programme, Ministry of Health Malaysia. The participants were also given the URLs of the national product and advertisement registration databases, where it is possible to check whether a product or advertisement has been registered with the authorities [42, 43]. No monetary reimbursement was provided to the participants.

## Results

A total of 18 IDIs were conducted with consumers with a median age of 34.5 years old (interquartile range: 31, 50 years old). The majority of the participants were female (72.2%) had a tertiary education level (88.9%) and had no comorbidity (77.8%). Some of the participants worked in the private sector (44.4%) and public sector (27.8%), while a few were self-employed (11.1%), retirees (11.1%) and housemaker (5.6%) (Table 1). The interviews lasted between 30 and 80 min.

**Table 1:** Socio-demographic characteristics of the participants

Participant	Sex^a^	Age in years	Ethnicity^b^	Educational level	Occupation	Health status^c^	Type of online product purchased
P 01	F	33	C	Tertiary	Civil servant	Healthy	Health supplement
P 02	F	29	C	Tertiary	Self-employed	Healthy	Health supplement
P 03	F	34	M	Tertiary	Civil servant	Healthy	Health supplement and natural product
P 04	M	30	C	Tertiary	Private employee	Healthy	Health supplement
P 05	F	66	C	Tertiary	Retired	NCD	Health supplement
P 06	F	30	M	Tertiary	Civil servant	Healthy	Health supplement
P 07	F	43	C	Tertiary	Housemaker	Healthy	Health supplement
P 08	F	31	C	Tertiary	Private employee	Healthy	Health supplement
P 09	F	50	C	Tertiary	Private employee	Healthy	Health supplement
P 10	M	35	M	Tertiary	Civil servant	Healthy	Natural product
P 11	F	24	I	Tertiary	Civil servant	Healthy	Health supplement
P 12	F	45	C	Tertiary	Private employee	Healthy	Health supplement
P 13	M	40	C	Tertiary	Private employee	Healthy	Health supplement
P 14	M	31	C	Tertiary	Private employee	Healthy	Health supplement
P 15	F	50	C	Secondary	Private employee	NCD	Natural product
P 16	F	32	C	Tertiary	Private employee	Healthy	Health supplement
P 17	M	74	C	Secondary	Self-employed	NCD	Health supplement
P 18	F	79	C	Tertiary	Retired	NCD	Health supplement

^a^*M* male, *F* female; ^b^*M* Malay, *C* Chinese, *I* Indian; ^c^*NCD* non-communicable disease

**Fig. 1:**
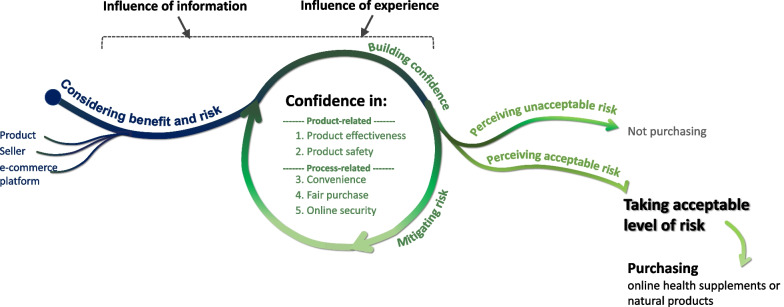
The substantive theoretical model explaining consumers’ behaviour in online purchasing of health supplements and natural products

A substantive theoretical model was developed to explain the considerations of consumers and the process undertaken prior to purchasing online health supplements and natural products. Consumers’ decisions when purchasing these products are the result of a series of assessments regarding the perceived benefits and risks of this activity, and these perceptions are constantly influenced by their experience in the activity and the information they have been exposed to (Fig. 1). When consumers perceive a product-related or process-related risk, they select products, online sellers, sales platforms and/or purchase mechanisms with lower perceived risk. As a result, consumers’ assessments and considerations of the benefits and risks are ultimately integrated into their confidence in five purchase-related elements: (1) product effectiveness, (2) product safety, (3) purchase convenience, (4) fair purchase and (5) online security. The first two elements relate to the product, and the latter three relate to the process.

Conceptually, consumer confidence based on these five elements and the associated risk perceived by the consumer are two sides of the same coin. When consumers perceive a significant risk that potentially negatively affects any of the five element(s) and cannot identify a better alternative with a lower risk, they may have insufficient confidence in the respective element(s), and their perceived risk is likely to be above their acceptable level. In conditions such as this, consumers are unlikely to carry out the online purchase of health supplements and natural products. In contrast, if consumers believe that a perceived risk is adequately overcome or mitigated by methods such as selecting a specific brand or purchasing from a specific online seller or platform, their confidence in those related element(s) increases, and the perceived risk is reduced to an acceptable risk level. Ultimately, consumers may take an acceptable level of risk to purchase a health supplement or natural product online.

### Confidence in the five elements of online health supplement and natural product purchasing

From the perspective of the consumer, perceived benefit refers to the ‘perception of relative advantages obtained from an acquisition when purchasing a product or service’; meanwhile, perceived risk is a ‘perceptual sense that any action consumers take will produce consequences they cannot anticipate’ [44]. For the purpose of this study, perceived benefit includes the advantages consumers expect to obtain from the activity of purchasing health supplements and natural products online, whereas perceived risk refers to a negative outcome or issue that consumers are concerned about or believe may occur as a result of purchasing health supplements and natural products online. Consumers consider the benefits and risks when they are in the process of reviewing the various options available in both the product-related and process-related domains before making a purchase. The product domain encompasses all aspects of the health supplements and natural products themselves, including the expected effects or side effects of these products. Conversely, the process domain comprises all the components related to the procedures involved during the online purchase, including product selection and payment through the online sales platform, product delivery to the consumer’s location, as well as customer services from the online seller.

**Fig. 2:**
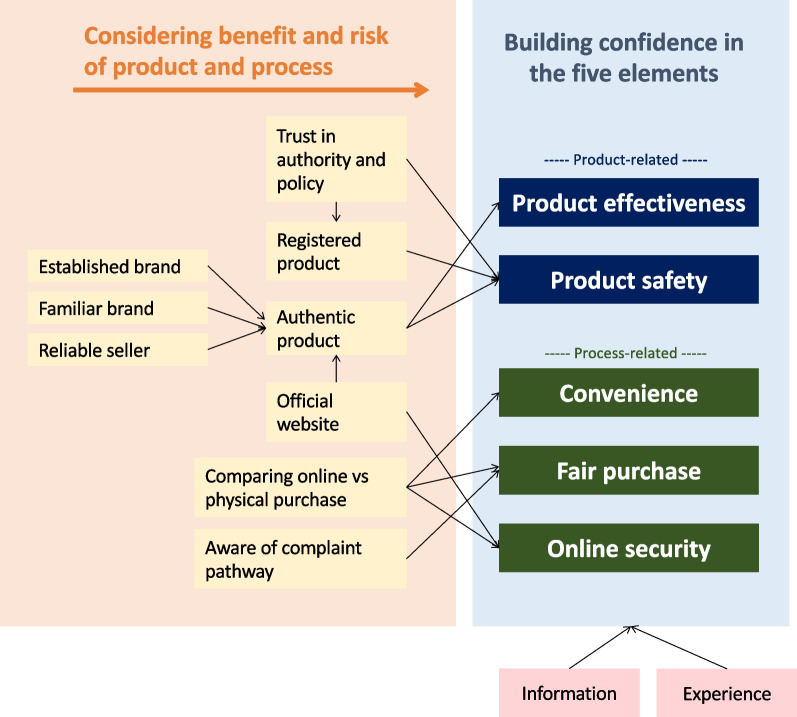
Consumers’ considerations regarding the product, online seller and e-commerce platform, which subsequently build their confidence in the five elements prior to the online purchase of health supplements and natural products

As a result of considering both the perceived benefits and perceived risks, consumers develop a certain level of confidence in both the product-related and process-related domains. Confidence is the feeling of the consumer that they can trust, believe in, and be certain of the good qualities of something [45], which, in the context of this study, is the product and the process. For any risk identified or perceived in relation to the online purchase of supplements, consumers make an effort to reduce that risk or overcome it. This behaviour is demonstrated when a consumer expresses a preference for a specific seller, company, or online platform. For instance, when consumers perceive that there is a higher risk of receiving fake products from individual sellers, they may opt to purchase from an ‘authentic seller’ (as claimed by the general online sales platform), as these sellers are perceived to have a lower risk of selling fake products. To ultimately engage in the activity of purchasing health supplements and natural products online, consumers must have sufficient confidence in the five elements relating to both the product and process domains: product effectiveness, product safety, process convenience, fair purchase and online security (Fig. 2). The level of confidence in each element varies with each consumer’s experience with the product and process, as well as the information they have gathered over time. Furthermore, the expectations for the five elements vary depending on consumers’ perceptions and the value they place on each element.

### Confidence in product effectiveness and product safety

To purchase a health supplement or natural product online, consumers must have sufficient confidence in the product’s effectiveness and believe that the product can help prevent disease, maintain health, or improve their health condition. The consumer must also believe that a product is safe in order to purchase it. Consumers’ emphasis on product effectiveness and safety is evident based on their concerns about product authenticity, which are related to the risks of experiencing ineffectiveness or unwanted side effects from counterfeit products or failing to gain the expected health benefits from it. Overall, consumers tend to purchase authentic products as they have greater confidence in their effectiveness and safety.

Although consumers cannot easily confirm the authenticity of an online product, they develop their confidence in the product’s authenticity through a variety of means, including opting for a product from an established brand, selecting an online product that they have previously seen in a physical pharmacy shop, or making an online purchase from a reliable company or seller. Consumers consider a product from a well-known brand to be safe because no negative events have been reported since its development. From the consumers’ perspective, if their product is unsafe, the company has *‘probably killed millions of people already’ (P13, 40-year-old healthy male).* Similarly, consumers also have confidence in the effectiveness and safety of an online product when that specific product is also available in a physical pharmacy, as one may *‘consider that he/she can trust everything that enters the pharmacy (physical pharmacy shop)’ (P06, 30-year-old healthy female)*. The consumers’ confidence in the established brand or the physical pharmacy shop is transferred to their perceptions of the quality of the respective online product.

Furthermore, consumers prefer to buy from online sellers that are perceived to be credible, as they have greater confidence in the effectiveness and safety of the products sold by those sellers. Online sellers are perceived to be more credible when they record more transactions and receive positive reviews from the customer in terms of their services and product quality. Furthermore, the label ‘authentic sellers’ given by online sales platforms is perceived as an indicator of a seller’s credibility by consumers, which, in turn, increases the consumers’ confidence in the products’ effectiveness and safety. One consumer in our study justified his action of purchasing health supplements from an ‘authentic seller’ by stating the following:

*‘…I need to make sure that, you know, the product that I’m buying or purchasing…is an authentic product. So, [the seller’s] reliability [has] a very high… value in my decision-making process.’*

*(P13, 40-year-old healthy male)*

Additionally, consumers prefer to purchase products from the official website in order to reduce the risk of receiving a fake or adulterated product over the Internet. The term ‘official website’ refers to the online website that is owned by the product company and represents that specific company on the Internet. When a consumer lacks trust in an individual seller on a general online sales platform, they may opt to buy health supplements or natural products from the official website of the product company, which they trust to a greater extent. As a result of purchasing from the official website, the consumers’ perceived risk of receiving a counterfeit or adulterated product is reduced, while their confidence in the product’s effectiveness and safety is increased. Similarly, consumers’ preference for purchasing health supplements and natural products from an official website is also an attempt to reduce the process-related risk (e.g., online payment fraud) and to increase their confidence in the respective process-related element of fair purchase. The following examples explain why consumers prefer to purchase from the official website rather than individual sellers:

*‘[I] will not [purchase supplements from the general online sales platform], because it is too risky for me, too many fake products. (Laughing) It is acceptable if the product is for external use, but if the product is for oral consumption, I feel that it is too risky.’*

*(P16, 32-year-old healthy female)*

Generally, the perception that a product has been registered with the authorities increases consumers’ confidence in the product quality and, particularly, the product safety. Consumers expect the local authorities to ensure their safety and, thus, believe that all registered products *‘have been checked by the Ministry of Health’ (P03, 34-year-old healthy female).* One consumer in our study expressed his preference for ensuring a product is registered with the health ministry prior to online purchase:

*‘...if I want to buy a supplement, I will look for [product registration with the authority]. That is one of my priorities.’*

*(P10, 35-year-old healthy male)*

It is worth noting that consumers’ confidence in a registered product is also based on their trust in the authority’s expertise. If consumers do not trust the authority’s capability to evaluate the product safety, the registration status of the product may not influence consumers’ confidence, as they may think that *‘probably the government also does not know how to certify them’ (P09, 50-year-old healthy female).* Furthermore, consumers’ trust in the local government’s policy for controlling online supplement sales influences their confidence in the effectiveness and safety of health supplements and natural products. Specifically, consumers have lower confidence in product safety when they perceive a lack of monitoring and regulation of online supplement sales by the authority, as *‘nobody verifies what they, [the online sellers], are selling’ (P02, 29-year-old healthy female).*

### Confidence in process convenience, fair purchase and online security

Firstly, to perform an online purchase, consumers must have confidence in the process’ convenience, meaning the process of product search, selection, order and transaction must be perceived to be feasible and manageable. Managing an online purchase, whether on a company website or a general online sales platform, is simpler for younger compared with older consumers. However, older consumers seek help from those they trust to assist them in making the online purchase, such as by *‘making a number of purchases initially under someone’s supervision’ (P18, 79-year-old female with NCDs).* This use of assistance in making purchases could increase the consumer’s confidence in the element of process convenience.

Notably, process convenience encompasses not only the ease of obtaining a product but also the ease of gathering personalised information that aids in the product search. The importance of being able to gather this information explains why some consumers prefer online purchases of health supplement and natural products, as these purchases allow for *‘easy and faster access to information’ (P11, 24-year-old healthy female)*. Indeed, this information can be obtained from an Internet search engine or a product review shared by a peer customer rather than by physically visiting different shops for comparison. However, other consumers prefer physical shop purchases in order to obtain product information directly from a shop employee; specifically, these consumers believe this process is more personalised and saves time as one *‘doesn’t need to do their homework (surveying about a product)’ (P02, 29-year-old healthy female).*

Secondly, a fair purchase is an important element that consumers consider prior to making an online supplement purchase, as they must feel that they have adequate freedom to make an unbiased decision in their choice of supplement when making a product purchase. Perceived conflicts of interest between the seller and the consumer often cause consumers to question whether the recommended product is appropriate for them, as they perceive that physical shop personnel *‘tend to push them towards supplements that they will get profit from’ (P11, 24-year-old healthy female).* Additionally, consumers are more confident in the fairness of a purchase when they understand how to file a product complaint or leave a negative review for an online seller if an unsatisfactory incident occurs. Similarly, some consumers prefer to purchase from the product company’s official website due to this giving them greater confidence in the fairness of the purchase. Indeed, purchasing from the official website means a specific company can be held accountable when a negative incident occurs, such as unsatisfactory product experiences or online payment failure:

*‘… if you are directly dealing with the company (purchase through the official website)... You have the receipt to prove that you bought it from them. If anything happens, touch wood, you can still deal with the company; this is a protection for yourself.’*

*(P16, 32-year-old healthy female)*

Thirdly, online security emerged as another process-related factor that consumers considered prior to purchasing online health supplements and natural products. Particularly, consumers expressed their concerns about data theft and financial loss due to disclosing personal and financial information during the process of online purchasing. One of our consumers expressed her preference to purchase health supplements and natural products from individual e-commerce platforms, particularly those belonging to an *‘internationally established company’*, because *‘the risk of personal information being hacked is lower’ (P08, 31-year-old healthy female)* compared to shared e-commerce platforms. Another consumer expressed great confidence in online security based on his understanding of the security measures in place for online payment mechanisms. This consumer explained:

*‘As long as you saw some big player in the market when you check out that time, for example, Razorpay, iPay88 [and] senangPay… all [these are] actually governed by a Paynet, which is a subsidiary of BNM (Bank Negara Malaysia, the Central Bank of Malaysia). So, I don’t think there’s a risk…’ (P14, 31-year-old healthy male)*

### Taking an acceptable level of risk in online purchases of health supplements and natural products—the core category

By definition, a person is said to be taking a risk when they do something that may result in loss or failure [46] in order to achieve something [47]. In the context of this study, the goal of the behaviour is to obtain health supplements or natural products, and the loss or failure that the consumer may experience refers to the negative consequences that may occur when obtaining these products online. The anticipation of these consequences constitutes the above-mentioned ‘risk’ that consumers perceive, which can be product- or process-related. It is worth noting that not all risk must be eliminated for consumers to purchase health supplements and natural products online. In particular, when a consumer is willing to accept a certain level of risk associated with this activity, this indicates risk-taking behaviour among consumers who buy health supplements and natural products online. For example, despite conducting extensive research and consideration prior to purchasing an online supplement, one consumer in our study still considered himself *‘pretty lucky’* because he didn’t have *‘any bad experience purchasing online’* thus far *(P13, 40-year-old healthy male).*

Taking some level of risk is deemed necessary for consumers to achieve the intended goal of enjoying the potential benefit of a supplement, because if the consumer is *‘suspicious or not willing to believe all the time, [they may] end up not buying [any supplements]…’ (P18, 79-year-old female with NCDs).* Therefore, consumers are willing to take a certain level of risk in exchange for the potential benefit they expect from the online purchase of health supplements and natural products. For instance, one consumer in our study took the risk of purchasing and taking an online supplement for the potential health benefits of the product despite the health risk she perceived from taking a supplement, which she considered ‘unnatural’ compared with eating fruits and vegetables. In this scenario, she took the risk of her health being harmed by a potentially unsafe product.

*‘I... I… really do not know the effects [of the supplements]. But I tell myself, take it (the supplements) for the peace of mind... I also tell myself these are not natural things.’ (P18, 79-year-old female with NCDs)*

Another example identified in our study involved a risk related to the process. Specifically, one consumer decided to make an online purchase despite being aware of the risk of reduced supplement quality due to improper delivery conditions. This is fairly common in the present world, where *‘time [constraint] is always [a] problem.’ (P18, 79-year-old female with NCDs).*

Consumers often perceive a greater risk during their initial attempt at purchasing health supplements or natural products online, when they encounter more uncertainties about new products, as well as when using a new online platform that they have no experience with. At these times, consumers put more effort into checking other consumers’ reviews, the seller’s credibility and the product quality to build confidence in the aforementioned five elements related to the purchase decision. The initial attempts at online supplement purchase represent a greater level of risk-taking behaviour by consumers, as they ‘*have to go for it, [as] only then can [they] prove [whether the online purchase is] feasible or not’ (P18, 79-year-old female with NCDs)*.

Furthermore, following the initial purchase attempts, ‘risk-taking’ behaviour persists for regular purchases. Although consumers require adequate confidence in the aforementioned five elements of the purchase decision, this does not mean that they do not perceive any risk in their subsequent online supplement purchases. Instead, in subsequent purchases, their perceived risk is mitigated to a lower level that they are comfortable with. For instance, one consumer in this study agreed that it was still possible for her to obtain fake products even though she had opted to purchase from an authentic online seller. However, she took the risk and made the online purchase after sufficient deliberation regarding the online seller from whom she intended to purchase the health supplement:

*‘...I’ve tried to filter, to buy it from an authentic seller, [or] an authentic shop, right? … So, I’ve tried to eliminate the risk of er... buying from an individual seller.’*

*(P03, 34-year-old healthy female)*

The acceptable level of risk refers to how much risk consumers can tolerate when deciding to purchase supplements online. This level is personal and is largely determined by the consumer’s perception of control over the potential consequences resulting from the risk. Having control over the consequences involves the consumer having the confidence to manage or handle the consequences or the impact of the consequences being acceptable for them. When consumers perceive they have this control, they perceive a lower risk in performing the activity and, thus, are more likely to purchase supplements online. For example, one consumer used ‘trial and error’ with health supplements purchased online in order to evaluate their effects. The term ‘trial and error’ demonstrates the consumer’s awareness of the possibility of ‘encountering errors’ in her trial, meaning the consumer perceived the risk of product ineffectiveness to be manageable.

*‘I [use] trial and error. For example, after taking [the supplement] for a while, I would stop and see, and then… [I will] resume taking [the supplement and] will feel the difference; this is how I test it’*

*(P12, 45-year-old healthy female)*

Furthermore, if consumers can mitigate the perceived risk to an acceptable level with perceived manageable consequences (e.g., purchasing an authentic product, using an authorised online sales platform to avoid receiving a fake product, or buying a smaller quantity to avoid financial loss), they are more likely to purchase supplements online.

On the contrary, when consumers lack confidence in dealing with the resulting consequences, or when the impact of the resulting consequences is perceived to be too detrimental (e.g., loss of privacy, harm to health due to serious adverse effects), consumers perceive a lack of control over the potential consequences. As a result, the perceived risk exceeds their acceptable level, thus reducing their willingness to purchase supplements online. In our study, one consumer explained that she would not risk purchasing and taking a health supplement if it caused palpitation, as this is a side effect that involves an important organ and that she perceives to be possibly fatal. The perceived risk was beyond her acceptable risk level, and, thus, she was unwilling to purchase and consume the online health supplements.

*‘… if we have general knowledge, we feel that it is abnormal for the heart to palpitate. If you experienced this (palpitation) every time you take the supplement, would you dare to take it?’*

*(P15, 50-year-old female with an NCD)*

Similarly, issues concerning the disclosure of personal information, including one’s home address, are associated with the risk of compromised personal safety, which could lead to detrimental consequences. In this study, one consumer expressed her perceived lack of control regarding the potential worst-case scenario, stating that if she *‘suddenly receive[s] [scam calls from fraudulent groups] … [she] will not be able to react’ (P08, 31-year-old healthy female).* Consequently, this consumer decided to refrain from making online purchases, as she found no alternative ways to mitigate the risk:

*‘In fact, there is nothing we can do about it (personal information security)... As a result, my strategy is to do less—reduce my purchasing frequency.’ (P08, 31-year-old healthy female)*

It is worth noting that, consumers’ willingness to take the risk of purchasing online health supplements or natural products could be influenced by their health conditions. For example, in our study, one participant with a history of liver disease was more cautious in his use of health supplements due to the possible harm from the inappropriate use of these products, as he felt obligated to *‘take care’* of his liver. *(P17, 74-year-old male with NCDs).* This participant’s willingness to take a risk when buying health supplements online is reduced by his medical history of liver disease. However, another participant claimed to be taking a health supplement because *‘it is supposed to be good for her macular degeneration’ (P18, 79-year-old female with NCDs).* This participant’s health condition most likely reinforces her willingness to take the risk despite her perception that health supplements, which are *‘made from chemicals’,* may be *‘not good’ for her health (P18, 79-year-old female with NCDs).*

Generally, ‘risk-taking’ behaviour is not limited to the purchase of health supplements and natural products on the Internet but also from physical shops. The following excerpt demonstrates that one consumer in this study perceived that there is a similar level of risk of receiving a fake product from a physical shop compared with online sales platforms:

*‘Because if I buy at a [physical] shop, I can’t really differentiate (identify a fake product), right? ... [but] we will still buy it, right? It’s the same.’ (P03, 34-year-old healthy female)*

Overall, the growing popularity of online shopping encourages online purchasing behaviour because a borderless online marketplace with a greater variety of supplements is available to the general public. Furthermore, the components of risk that consumers need to manage when purchasing health supplements or natural products online may differ from those related to purchasing these products from physical shops.

## Discussion

A substantive theoretical model has been developed to explain consumers’ behaviour when purchasing health supplements and natural products over the Internet. The model reveals that consumers’ decisions when making online purchases of supplements and natural products are made based on adequate confidence in both the product (i.e. product effectiveness, product safety) and the process of purchasing online (i.e. convenience, fair purchase, online security). Ultimately, ‘taking an acceptable level of risk’ was found to be the key concept in the process of consumer purchases of health supplements and natural products from the Internet. Consumers’ perceptions and priorities regarding the product and process are personal and are also influenced by information from various sources as well as their experience using and purchasing the product.

This study provides valuable insight by conceptualising a substantive theoretical model that encompasses both health behaviour in the use of health supplements and the use of technology in the acquisition of the products. Additionally, the theoretical model includes five priorities on which consumers base their selection of products and online platforms, and these five elements provide policymakers with a better understanding of consumers’ underlying considerations during online purchases of health supplements and natural products. The HBM lists a total of six constructs that predict health behaviour in individuals, including perceived susceptibility, perceived severity, perceived benefit, perceived barriers, cues to action and self-efficacy [48]. The five elements in the model formed by this study provide further contextual details to understand consumers’ ‘perceived benefit’ and ‘perceived barriers’ prior to the online purchase of health supplements and natural products. Moreover, the PMT is another theory that is frequently used to discuss individuals’ motivations to protect their health based on threat and coping appraisals [24, 25]. An individual must perceive their susceptibility to a disease and the severity of a disease (threat appraisal) to consider taking a measure to prevent it. Additionally, the individual should also perceive the measure to be effective (response efficacy), perceive that they can adopt that measure (self-efficacy) and perceive that the measure has a reasonable response–cost relationship (cost efficacy), which includes any cost of taking the measure, such as inconvenience and side effects [49]. In the context of online purchases of health supplements and natural products, this study highlights that consumers ‘take an acceptable level of risk’ when performing these purchases. This result provides valuable insight into the approach that consumers take after estimating the perceived risk of an online supplement purchase, adding valuable detail to the existing concept of ‘response cost’ articulated by the PMT.

The core concept that emerged from this study is that consumers ‘take an acceptable level of risk’ to arrive at their decision to purchase online health supplements and natural products. Human risk-taking behaviour has been studied in a variety of contexts, including in relation to social, health, safety, financial, ethical and recreational aspects [50]. Risk-taking behaviour is part of the decision-making process when there is uncertainty or unpredictability in a situation [51], such as when a consumer is unsure about the quality of an online health supplement or the safety of an online transaction. Additionally, risk-taking behaviour can also be explained as a trade-off between return and risk [50]. In the context of this study, the behaviour of ‘taking an acceptable level of risk’ could be viewed as a trade-off between the perceived risk and perceived benefit, either related to the product or the process. For instance, consumers may trade off process convenience for uncertainty about the product quality or could trade off personal data security for the potential health benefits of the health supplement.

Regarding the theoretical model, the consideration of benefits and risks prior to online purchases of health supplements and natural products is based on the consumers’ perceptions, which may be different from the reality. Consumers’ perceptions are their construction of meaning and reality. Indeed, studies have reported that not every consumer understands how health supplements work despite providing them to children in their care [52], as well as that not every consumer is aware of online privacy regulations despite using online shops [53]. These mismatches between consumers’ perceptions of risk and the reality tend to impede consumers’ accurate risk stratification. In turn, such inaccurate situational assessments may lead to perceptual biases in decision-making, which may ultimately affect individuals’ risk-taking behaviour [51]. Therefore, it is critical to ensure that consumers are informed about the potential benefits and risks associated with both products and the purchasing process in order to support them to avoid risk by making informed purchasing decisions.

Five elements were outlined to describe consumers’ priorities in considering their online purchases of health supplements and natural products. The concept of ‘confidence’ has been widely studied, and confidence is commonly regarded as an important factor for predicting consumers’ decisions [54–56]. Focusing on the product-related domains, product effectiveness and product safety are other factors that consumers consider when making an online purchase, as an unsafe product would have a negative impact on their health. The notion of ‘product safety’ illustrates consumers’ desire to protect their health while purchasing health supplements and natural products online, highlighting the distinct contributions of this substantive theoretical model over previous models. A ‘lower product price’ is commonly perceived as a significant benefit of online purchases [44, 52, 57, 58]. However, a health supplement or natural product with an excessively low price may make consumers suspicious of the product’s authenticity and, thus, the product effectiveness and safety, revealing the differences in consumers’ priorities across different types of products.

In terms of the process-related domains, consumers consider the convenience of the purchase mechanism, the fairness of the purchase and online security after performing the online purchase. Similar to the existing literature [59, 60], this study found that consumers construct the convenience of online purchases based not only on how easy it is to purchase a product but also on other dimensions, such as the ease of searching, accessing and comparing the features and prices of various products. Notably, although a lack of product tangibility in the online purchase environment limits consumers’ ability to evaluate the product quality and, thus, deters them from making online purchases [55, 61], this lack of tangibility is unlikely to be a major concern with health supplements and natural products, as their quality cannot be assessed prior to purchase [62].

Moreover, consumers’ concerns about the fairness of a purchase reflects the interpersonal aspect of consumer expectation, which refers to the fact that consumers need to be confident that they are being honestly and fairly treated by the seller in order to complete the online purchase [63]. In this study, the consumers perceived the absence of physical shop staff in the virtual marketplace as a benefit. Indeed, this absence gives consumers autonomy over their decision without being ‘pushed’ by shop staff to purchase a product that may not be the best fit for them, which would not be ‘fair’ to them. Although consumers prefer a pressure-free purchase experience, which has been reported even in physical settings [64], this absence of staff also means that consumers purchase products without advice from a salesperson or pharmacist. The potential dangers and consequences of such unguided purchases of health supplements and natural products on e-commerce platforms deserve more attention from healthcare authorities.

Finally, online security is also a concern of consumers, and this stems from a sense of vulnerability due to the need to disclose personal information such as their phone number, bank account details and address [56]. The compromise of this personal information could result in significant financial losses for the consumer. This concern is likely to be similar in the online purchase of products other than health supplements and natural products. This study adds to the picture by highlighting consumers’ concerns about personal safety, in addition to financial safety, as a result of the need to disclose personal contact information during the online purchase process. This finding is likely to support the findings of another study, which found that consumers impose stricter protection methods when dealing with their personal contact information in the online environment, as opposed to other information about their social identity and daily life [65]. Besides, consumers’ concern for personal safety also reflects their concern for how the virtual activity may influence their physical life. The security of the online environment, whether it is related to consumers’ personal safety or financial safety, is thus a critical issue that requires attention from e-commerce providers and authorities in order to ensure consumers' confidence in online purchases.

### Policy implications of the study

The study’s findings raise concerns about consumer behaviour in the online purchase of health supplements and natural products, which is becoming more popular in current the information technology era. The public’s acceptance of online purchases implies that online purchases have been adopted as an alternative to physical shop purchases for health supplements and natural products. Furthermore, this acceptance shows that the general public is now exposed to and must manage different risks when purchasing supplements online compared to in a physical shop. Therefore, a more controlled online marketplace is required with regard to the sales of health supplements and natural products.

However, the quality control and regulation of health supplements and natural products are always difficult. Furthermore, the rise of e-commerce has magnified the problems with regulating and monitoring the sales and use of these products among the general public, as the online environment is currently relatively unregulated, and consumers must make the best decisions for themselves. International cooperation in the control of online sales of health supplements and natural products should be increased in order to develop effective policy regulation. Awarding accreditation to reliable sellers who have been assessed in terms of their product supply chain, storage conditions and delivery procedures would allow consumers to be informed about where they can source high-quality and safe health supplements and natural products.

It is of utmost importance to provide consumers with adequate information regarding product and process safety to enable informed decision-making. Great effort has been taken by the National Pharmaceutical Regulatory Agency (NPRA) of the Malaysian Ministry of Health to organise educational brochures and product safety information for public use [66], including lists of registered products (available on the ‘Quest 3 + product search’ website) [43], as well as adulterated and banned products. This website also allows consumers to report any suspected side effects or unwanted effects from any product, including health supplements and natural products. The use of this website by consumers should be continually advocated, and the content should be proactively disseminated to consumers to attract the attention of the public. Finally, consumers should be encouraged and educated to notify the authorities if any adverse effects occur as a result of using any health supplement or natural product.

### Strengths and limitations of the study

At the initial phase of the study, purposive sampling of participants from various demographic backgrounds ensured that we captured significant variation in consumer experiences. In addition, the adoption of the grounded theory approach enabled the study to generate a substantive theoretical model based on empirical data, which reflects consumers’ behaviour when purchasing health supplements and natural products online in the real world. Additionally, the grounded theory approach incorporates several steps that ensure the quality of the study findings [67]. For example, concurrent data collection and analysis allows categories to be identified from the data rather than the data being forced into the categories. Furthermore, the theoretical sampling of participants with diverse medical histories did not result in the addition of new categories to the emerging theoretical model, indicating theoretical saturation. Finally, writing memos throughout the study ensured the analysis was grounded in the data and served as a method of controlling the researcher’s personal bias.

It is worth noting that the public’s perceptions and beliefs about the use of health supplements and natural products differ depending on cultural background [68–70], as do consumer behaviours [71]. As this study was conducted among Malaysians, an Asian population, the results should be interpreted with caution when applied to other populations. Furthermore, this study focused on health supplements and natural products that are taken orally rather than topically. Given that some consumers also perceive topical products to be health supplements, one should exercise caution when interpreting the findings in the context of health supplements and natural products with other routes of administration. Nonetheless, the emerging theoretical model resonates with information that has been reported in the literature and other theoretical models, implying that the study findings are potentially transferable to other populations in the context of online purchasing of health supplements and natural products.

## Conclusions

This study provides a substantive theoretical model that highlights consumers’ considerations when making online purchases of health supplements and natural products in both product-related and process-related domains: (1) product effectiveness, (2) product safety, (3) purchase convenience, (4) fair purchase and (5) online security. The model suggests that consumers ultimately take an acceptable level of risk to make the decision to purchase health supplements and natural products online. Furthermore, the model shows that individual perceptions of benefit and risk vary and are constantly influenced by the information and experience to which the individual is exposed. This substantive model is potentially transferable to other populations.

## Data Availability

All data generated or analysed during this study are included in this published article.

## Abbreviations

HBM: Health belief model
HIPAA: Health Insurance Portability and Accountability Act
IDI: In-depth interview
NPRA: National Pharmaceutical Regulatory Agency
PMT: Protection motivation theory

## References

[1] Hys K. Identification of the reasons why individual consumers purchase dietary supplements. In: Sroka W, editor. Perspectives on consumer behaviour. contributions to management science. Switzerland: Springer Nature; 2020. p. 193–209.

[2] Rautiainen S, Manson JE, Lichtenstein AH, Sesso HD (2016). Dietary supplements and disease prevention—a global overview. Nat Rev Endocrinol.

[3] Peltzer K, Pengpid S (2015). Utilization and practice of traditional/complementary/alternative medicine (T/CAM) in Southeast Asian Nations (ASEAN) member states. Ethno Med.

[4] Mohd Zaki NA, Rasidi MN, Awaluddin SM, Hiong TG, Ismail H, Mohamad Nor NS (2018). Prevalence and characteristic of dietary supplement users in Malaysia: data from the Malaysian Adult Nutrition Survey (MANS) 2014. Glob J Health Sci.

[5] Bowman C, Family H, Agius-Muscat H, Cordina M, Sutton J (2020). Consumer internet purchasing of medicines using a population sample: a mixed methodology approach. Res Social Adm Pharm.

[6] Brijnath B, Antoniade J, Adams J (2014). Investigating patient perspectives on medical returns and buying medicines online in two communities in Melbourne, Australia: results from a qualitative study. Patient.

[7] Jairoun AA, Al‑Hemyari SS, Mohammed Abdulla N, El‑Dahiyat F, Jairoun M, AL‑Tamimi SK, et al. Online medication purchasing during the Covid‑19 pandemic: a pilot study from the United Arab Emirates. J Pharm Policy Pract. 2021;14(38):1–7. 10.1186/s40545-021-00320-z. 10.1186/s40545-021-00324-9 PMC8111048 33975632

[8] Eger L, Komarkov L, Egerov D, Micík M (2021). The effect of COVID-19 on consumer shopping behaviour: generational cohort perspective. J of Retailing and Consumer Services.

[9] Liu C, Sun CK, Chang YC, Yang SY, Liu T, Yang CC (2021). The impact of the fear of COVID-19 on purchase behavior of dietary supplements: integration of the theory of planned behavior and the protection motivation theory. Sustainability.

[10] Chiba T, Tanemura N (2022). The prevalence of dietary supplement use for the purpose of COVID-19 prevention in Japan. Nutrients.

[11] UNCTAD. Covid-19 and e-commerce: a global review: United Nation Publications; 2021.

[12] Amos C, Pentina I, Hawkins TG, Davis N (2014). “Natural” labeling and consumers’ sentimental pastoral notion. J of Product & Brand Management.

[13] Kwan D, Beyene J, Shah PS (2009). Adverse consequences of internet purchase of pharmacologic agents or dietary supplements. J Pharm Technol.

[14] Ronis MJJ, Pedersen KB, Watt J (2018). Adverse effects of nutraceuticals and dietary supplements. Annu Rev Pharmacol Toxicol.

[15] Ekor M (2014). The growing use of herbal medicines: issues relating to adverse reactions and challenges in monitoring safety. Front Pharmacol.

[16] Asher GN, Corbett AH, Hawke RL (2017). Common herbal dietary supplement–drug interactions. Am Fam Physician.

[17] Fasinu PS, Bouic PJ, Rosenkranz B (2012). An overview of the evidence and mechanisms of herb–drug interactions. Front Pharmacol.

[18] Binns CW, Lee MK, Lee AH (2018). Problems and prospects: public health regulation of dietary supplements. Annu Rev Public Health.

[19] Thakkar S, Anklamv E, Xu A, Ulberth F, Li J, Li B (2020). Regulatory landscape of dietary supplements and herbal medicines from a global perspective. Regul Toxicol Pharmacol.

[20] Tripathi C, Girme A, Champaneri S, Patel RJ, Hingorani L (2020). Nutraceutical regulations: an opportunity in ASEAN countries. Nutrition.

[21] Chung JE, Stoel L, Xu YJ, Ren J (2012). Predicting Chinese consumers’ purchase intentions for imported soy-based dietary supplements. British Food Journal.

[22] Tzeng SY, Ho TY (2022). Exploring the effects of product knowledge, trust, and distrust in the health belief model to predict attitude toward dietary supplements. SAGE Open.

[23] Baranowski T, Cullen KW, Baranowski J (1999). Psychosocial correlates of dietary intake: advancing dietary intervention. Annu Rev Nutr.

[24] Henson S, Masakure O, Cranfield J (2008). The propensity for consumers to offset health risks through the use of functional foods and nutraceuticals: the case of lycopene. Food Qual Prefer.

[25] Cox DN, Koster A, Russella CG (2004). Predicting intentions to consume functional foods and supplements to offset memory loss using an adaptation of protection motivation theory. Appetite.

[26] Heijden HVD, Verhagen T, Creemers M (2003). Understanding online purchase intentions: contributions from technology and trust perspectives. Eur J Inf Syst.

[27] Mishra D, Akman I, Mishra A (2014). Theory of reasoned action application for green information technology acceptance. Comput Human Behav.

[28] Ha S, Stoel L (2009). Consumer e-shopping acceptance: antecedents in a technology acceptance model. J Bus Res.

[29] Tang H, Rasool Z, Khan MA, Khan AI, Khan F, Ali H (2021). Factors affecting e-shopping behaviour: application of theory of planned behaviour. Behav Neurol.

[30] Hsu MH, Chang CM, Chu KK, Lee YJ (2014). Determinants of repurchase intention in online group-buying: the perspectives of DeLone & McLean IS success model and trust. Comput Human Behav.

[31] Valvi AC, Frangos CC, Frangos CC (2013). Online and mobile customer behaviour: a critical evaluation of grounded theory studies. Behav Info Technol.

[32] National Pharmaceutical Regulatory Agency, Ministry of Health, Malaysia. Drug registration guidance document (DRGD), Appendix 6—guideline on registration of health supplements. 3rd ed; 2022.

[33] National Pharmaceutical Regulatory Agency, Ministry of Health, Malaysia. Drug registration guidance document (DRGD), Appendix 7—guidance on registration of natural products. 3rd ed; 2022.

[34] Charmaz K. Constructing grounded theory: a practical guide through qualitative analysis. Silverman D, editor: Sage Publication Ltd; 2006.

[35] Shakirah MS, Ang ZY, Anis Syakira J, Cheah KY, Kong YL, Selvarajah S (2020). The COVID-19 chronicles of Malaysia.

[36] Tates K, Zwaanswijk M, Otten R, Dulmen SV, Hoogerbrugge PM, Kamps WA (2009). Online focus groups as a tool to collect data in hard-to-include populations: examples from paediatric oncology. BMC Med Res Methodol.

[37] Lobe B, Morgan D, Hoffman KA (2020). Qualitative data collection in an era of social distancing. Int J Qual Methods.

[38] Birbili M. Translating from one language to another. Soc Res Update. 2000(31). https://sru.soc.surrey.ac.uk/SRU31.html.

[39] Nes FV, Abma T, Jonsson H, Deeg D (2010). Language differences in qualitative research: is meaning lost in translation?. Eur J Ageing.

[40] Nurjannah I, Mills J, Park T, Usher K (2014). Conducting a grounded theory study in a language other than English. SAGE Open.

[41] Larkin PJ, Casterle BDD, Schotsmans P (2007). Multilingual translation issues in qualitative research: reflections on a metaphorical process. Qual Health Res.

[42] Pharmaceutical Services Programme, Ministry of Health, Malaysia. Advertisement approved by Medicine Advertisements Board. 2022. https://www.pharmacy.gov.my/v2/en/apps/iklan. Accessed 3 Nov 2022

[43] National Pharmaceutical Regulatory Agency, Ministry of Health, Malaysia. Quest 3+ product search. https://quest3plus.bpfk.gov.my/pmo123xyz/. Accessed 3 Nov 2022

[44] Lim WM (2020). An equity theory perspective of online group buying. J Retail Consumer Serv.

[45] Oxford University Press. Definition of confidence noun from the Oxford Advanced American Dictionary 2022. https://www.oxfordlearnersdictionaries.com/definition/american_english/confidence#:~:text=noun-,noun,have%20confidence%20in%20their%20manager. Accessed 3 Nov 2022.

[46] Merriam-Webster, Incorporated. Merriam-Webster dictionary 2022. https://www.merriam-webster.com/dictionary/take%20a%20risk. Accessed 3 Nov 2022.

[47] Oxford University Press. Definition of risk-taking noun from the Oxford Advanced Learner's Dictionary 2022. https://www.oxfordlearnersdictionaries.com/definition/english/risk-taking?q=taking+risk. Accessed 3 Nov 2022.

[48] U.S. Department of Health and Human Services, National Institutes of Health, National Cancer Institute. Theory at a glance: a guide for health promotion practice. 2nd ed, 2005.

[49] Rogers RW, Cacioppo JT, Petty RE. Cognitive and physiological processes in fear appeals and attitude change: a revised theory of protection motivation: Guilford; 1983

[50] Figner B, Weber EU (2011). Who takes risks when and why? determinants of risk taking. Value in Health.

[51] de-Juan-Ripoll C, Giglioli IAC, Llanes-Jurado J, Marín-Morales J, Alcañiz M. Why do we take risks? Perception of the situation and risk proneness predict domain-specific risk taking. Front Psychol. 2021;12:562381. doi:10.3389/fpsyg.2021.562381. 10.3389/fpsyg.2021.562381 PMC7982407 33762988

[52] Liu HM, Zhang SY, Zou HS, Pan YL, Yang QP, Ouyang YF (2019). Dietary supplement use among chinese primary school students: a cross-sectional study in Hunan province. Int J Environ Res Public Health.

[53] Turow J, Hennessy M, Bleakley A (2008). Consumers' understanding of privacy rules in the marketplace. J Consum Aff.

[54] Choi CJ, Eldomiaty TI, Kim SW (2007). Consumer trust, social marketing and ethics of welfare exchange. J Bus Ethics.

[55] Oghazi P, Karlsson S, Hellström D, Hjort K (2018). Online purchase return policy leniency and purchase decision: mediating role of consumer trust. J Retail Consumer Serv.

[56] Harridge-March S (2006). Can the building of trust overcome consumer perceived risk online?. Mark Intell Plan.

[57] Arora N, Aggarwal A (2018). The role of perceived benefits in formation of online shopping attitude among women shoppers in India. South Asian J Bus Stud.

[58] Gurau C (2005). Pharmaceutical marketing on the internet: marketing techniques and customer profile. J Consum Mark.

[59] Duarte P, Silva SCE, Ferreira MB (2018). How convenient is it? delivering online shopping convenience to enhance customer satisfaction and encourage e-WOM. J Retail Consumer Serv..

[60] Jiang LA, Yang ZL, Jun MJ (2013). Measuring consumer perceptions of online shopping convenience. J of Service Management.

[61] Kamalul Ariffin S, Mohan T, Goh YN (2018). Influence of consumers’ perceived risk on consumers’ online purchase intention. JRIM.

[62] Leahy AS (2005). Search and experience goods: evidence from the 1960’s and 70’s. JABR..

[63] Chen YT, Chou TY (2012). Exploring the continuance intentions of consumers for B2C online shopping: perspectives of fairness and trust. Online Inf Rev.

[64] Bäckström K, Johansson U (2017). An exploration of consumers’ experiences in physical stores: comparing consumers’ and retailers’ perspectives in past and present time. Int Rev Retail Distrib Consum Res.

[65] Xie W, Karan K (2019). Consumers’ privacy concern and privacy protection on social network sites in the era of big data: empirical evidence from college students. J Interact Advert.

[66] National Pharmaceutical Control Bureau MoH, , Malaysia. Consumer main page. https://npra.gov.my/index.php/en/consumers. Accessed 3 Nov 2022.

[67] Elliott N, Lazenbatt A (2004). How to recognise a ‘quality’ grounded theory research study. Aust J Adv Nurs.

[68] Kofoed CLF, Christensen J, Dragsted LO, Tjønneland A, Roswall N (2015). Determinants of dietary supplement use – healthy individuals use dietary supplements. Br J Nutr.

[69] Akhagba OM (2017). Cultural influence in the consumption of herbal medicine among Nigerian women: a theoretical exploration. Miscell Anthropol Sociol..

[70] Guenther E, Mendoza J, Crouch BI, Moyer-Mileur LJ, Junkins EPJ (2005). Differences in herbal and dietary supplement use in the Hispanic and non-Hispanic pediatric populations. Pediatr Emerg Care.

[71] Mooij MD, Hofstede G (2011). Cross-cultural consumer behavior: cross-cultural consumer behavior: a review of research findings. J Int Consum Mark.

